# Supplementary material for: Arabidopsis Toxicos en Levadura 12 Modulates Salt Stress and ABA Responses in Arabidopsis thaliana
Source: Int J Mol Sci. 2022 Jun 30;23(13):7290. doi: 10.3390/ijms23137290 (PMC9266925; doi:10.3390/ijms23137290)
Supplement: Supplementary file 1 [file ijms-23-07290-s001.zip › Supplemental Figure S1.pdf]

```
Zea                1 ------------------------------------------------------------
Oryza              1 ------------------------------------------------------------
S.italica          1 ------------------------------------------------------------
Solanum            1 ------------------------------------------------------------
Sesamum            1 ------------------------------------------------------------
Gossypium          1 ------------------------------------------------------------
B.napus            1 ------------------------------------------------------------
AtATL43            1 ------------------------------------------------------------
O.                 1 ------------------------------------------------------------
S.bicolor          1 ------------------------------------------------------------
Setaria            1 ------------------------------------------------------------
Phaseolus          1 --------------------------------------MHWSI-----------------
Populus            1 MLMVREIGLSLAILMREKQTFAPYNKKLKIPYHPVCSLAKWAKLPFSSSALKIDAPLDLI
S.lycopersicum     1 ------------------------------------------------------------
Brassica           1 ------------------------------------------------------------
AtATL42            1 ------------------------------------------------------------
Arabidopsislyra    1 ------------------------------------------------------------
BrassicaNapus      1 ------------------------------------------------------------
Arabidopsis        1 ------------------------------------------------------------
A.lyrata           1 ------------------------------------------------------------
C.sativus          1 ------------------------------------------------------------
Cucumis            1 ------------------------------------------------------------
Medicago           1 ------------------------------------------------------------
Glycine            1 ------------------------------------------------------------
consensus          1

Zea                1 --------MEP------------------------ARRLLLSD-------------YDGA
Oryza              1 --------MEP------------------------SRRLLLSD-------------YDGA
S.italica          1 -----------------------------------------------------------M
Solanum            1 -----MNIYNVYFL-------------SLSL-WLLMITVILNAQ--------------EI
Sesamum            1 ---------MGVSG-------------LLSF-F--CFCVFLICEIAVATET-----AANS
Gossypium          1 -----MGLSYPSLP-------------FFLP-ILFFIPLLFADNYTNYYNTNNNNNNNGN
B.napus            1 --------MSSS---------------SSLL-LVSLLSLFLNASLADNDRYNTTAVVIMN
AtATL43            1 --------MSSS---------------SLIL-LFSTLSLFLNVSLADNHT----AVVITT
O.                 1 -----MGAVSMSTT------------TRLPLVAALLSAAACVAA--------------AQ
S.bicolor          1 --------MALPVP------------GQQLALLAVLFSAAAVAA--------------AQ
Setaria            1 --------MALPRQ------------LFLPLLAVVLSAAVCSAA--------------AQ
Phaseolus          6 -----------ITGGMISIFRNMPFKNHVPV-FIMMGLSFISNV--------------QA
Populus           61 HIVTFRDPLAFPRAGYIQSVAISMN--RL-L-LIIILTLFFHVK--------------AQ
S.lycopersicum     1 --------------------MR-----KN-I-LMLLLLSLNVKA--------------E-
Brassica           1 ---------------------MYQI--FI-F-FLTIFHSYYYAS--------------AQ
AtATL42            1 ---------------------MYQI--FF-F-FLPLLHSYAS----------------AQ
Arabidopsislyra    1 ---------------------MYQI--FF-F-FLPLLHSYPHVY--------------AQ
BrassicaNapus      1 --------MNPPKA------ISTLF-----F-SIFFFLDYVSAQ---------------S
Arabidopsis        1 --------MNSPQE------ISILF--FF-----IIFLDYVSA-----------------
A.lyrata           1 --------MNSPQE------ISILF--FF-F-FIIIFLDYVSAQ---------------S
C.sativus          1 ------------------------------------------------------------
Cucumis            1 ---------------------MIQL--SL-N-VCFLFSVFFVVE--------------AQ
Medicago           1 --------------------MMNQF--GI-T-LLILSTLFFHIR--------------AQ
Glycine            1 --------------------MNQLC--GV-I-LLVHSVLCFHAR--------------AQ
consensus         61                                  . .   .                  ..
```

```
Zea                16  IESPLPS-PP---------PS--SATPFRPGVAVV--VGILTSVFSITFLLLLYAKHCKRS
Oryza              16  IVSPLPS-PP---------ASSSAATPFRPGVAVV--VGILTSVFSITFLLLLYAKHCKRS
S.italica           2  TATPMAPSPP-----AARSPPPLPPPLAVDVAVI--AGVLTAVLLALFLFLIYAKHCKHR
Solanum            28  AESPSPSPPP---------YYRSRGSPFRPSIVIV--VAILTTIFSIVFMLLLYAKHCKRE
Sesamum            31  THSPVAPPPP-----AAAFDGAIANAFKPSIVIV--VGILTTIFSITFLLLLYVKHCKRE
Gossypium          42  TNFSTNPFPS-----PSQTKPNSSSPFKPSTAVVAFVAILTTLFAIIFLLLLYSKHCGGD
B.napus            37  PTTPPPLPPP-----SPLQRHNVTSSFMPGIAVV--IAVLTVVFSLTFLLLLYVKHCKRR
AtATL43            33  SDTPPPLPPP-----SPPPRHNFTSSLMPGIAVV--IAVLTAFFSLTFLLLLYVKHCKRR
O.                 30  PATAEAAPE---------TPGVGIKVSFRPSVAIV--VGIFTMIFSLTFLLLMYAKFCHPS
S.bicolor          27  PTTDELSASPAADNNSAPAGVGIKVSFRPRVAIV--VGIFTMIFSLTFILLMYAKFCHPS
Setaria            27  PTTAEAAPDNN-----STPGAGIKVSFRPSVAIV--VGIFTMIFSLTFLLLMYAKFCHPS
Phaseolus          40  QYSE-------------TDTMQDFPQPVRPSKMVV--IVALSILFTISFILLVYIRFCRTT
Populus           103  IT---------------SGSDSATVIPVHQGLPLI--LGILSTMLLITFFVLAYAKYCGRN
S.lycopersicum     19  -DLP-------------TYSSQDVVSSFQPSLAVV--IVVLFIMFSLTFVILFYAKFCHRT
Brassica           22  PPAP-------------PFRNGDLVTNFEPSLAVV--TGVLAIMFTLTFVLLVYAKCCHID
AtATL42            20  -TPP-------------PFRNGDLVANFEPSLAVV--TGVLAIMFALTFVLLVYAKCCHID
Arabidopsislyra    22  -PPP-------------PFRNGDLVANFEPSLAVV--TGVLAIMFALTFVLLVYAKCCHID
BrassicaNapus      26  -PPP-------------PPFLYAANGLFQPSLAII--TGVFSIVFTLTFVLLIYAKCFHND
Arabidopsis        23  QSPP-------------PPNLYATSDLFKPSLAII--TGVFSIVFTLTFVLLVYAKCFHND
A.lyrata           28  PPPP-------------PPDIYATSDLFKPSLAII--TGVFSIVFTLTFVLLVYAKCFHND
C.sativus           1  MD-----------------SQDAENSAFQPSLGFV--IGILGVMFLLTFILLVYAKFCHRR
Cucumis            22  ID-----------------SQDAENSAFQPSLGFV--IGILGVMFLLTFILLVYAKFCHRR
Medicago           23  SPTQ-------------TTNSQDAVSNFQPSLVVV--IAILGLMFSLTFILLIFAKVCHRR
Glycine            23  TA----------------APSGDAVSNFQPSLAVV--IGILGVMFLLTFFLLMYAKFCQRC
consensus         121    ..                      . ........  ...........*..*.... ...
consensus           1

Zea                63  AAESSGPYG----SAGSGGGFGSSGNAGGDRRNSGVDRAVVESLPVFRFGALRGQK-EG
Oryza              65  AAESSGPYG----GGGASGGA-------AGERRNSGVDRAVVESLPVFRFGALRGQK-AG
S.italica          55  GGAGAGGGG----RPGLGLGFAPS----SCERCRSGLSGSAVGALPAVRFGDMAGAG-AG
Solanum            78  GEFGMTG----------G-GGGLTNAGSSSFRKNSGIDRTVIESLPVFRFGSLRGQKAEG
Sesamum            84  IYESSNS----------R-GR-------PLSRGNSGINRKVIESLPMFRFSSLMGQK-DG
Gossypium          97  NDISNYNTT---NP-------FSYSSSLVTAGRNSGINRVVVESLPLFRFSSLSGHK-NG
B.napus            90  NGYVNQTQRFATSHGG-------GYGGVGVGRKNSGIDRSVIESLPVFRFGALSGHK-EG
AtATL43            86  NGSVYVNH-PQRFAITRYGGGYYNGGGVVGGRKNSGIDRSVIESLPVFRFGALSGHK-DG
O.                 80  SPVAAPAPT-----------------VVPAAAADDGVAKPVIESLPFFRFAALRGAR-QG
S.bicolor          85  SSP-SPAASALVGGG----ETGGNNG-AAAAAAAVGVPKQVIESLPFFRFATLRGAR-QG
Setaria            80  SSSPLPAAIPTAAAA----AAGNDAAAAALAPAQAGVPKPVIEALPFFRFAALRGAR-QG
Phaseolus          86  PLELINRRNLHS----------PNFQALTQSRSRSSGIDKKVIEALPFFMFSSLKGSK-QG
Populus           147  QNNFLGRYLHHQ----------NFHGLIRSSSRFSGIGEEVINSMPFFRFSSLKGSK-EG
S.lycopersicum     64  SSDDGGQLVRSV------------------------------------------------
Brassica           68  LRSGTGDGRRQDRRLR---QGIFFNRSTNSSDRFSGLDKTAIESLPLFRFSALKGSK-QG
AtATL42            65  LRSGSGDRRRHDRRLR---QGIFFNRSTASSDRFSGLDKTAIESLPLFRFSALKGSK-QG
Arabidopsislyra    67  LRSDSGDRRRHDRRLR---QGIFFNRSTTSSDRFSGLDKTAIESLPLFRFSALKGSK-QG
BrassicaNapus      71  LRSETYEDGEIRRL------DRLWQGLFSQSSRLSGLDKTAIESLPFFRFSALKGSK-QG
Arabidopsis        69  LRSETDSDGERIRH------DRLWQGLFNRSSRFSGLDKKAIESLPFFRFSALKGLK-QG
A.lyrata           74  LRSETDGDGETRRH------DRLWQGLFNRSSRFSGLDKKAIESLPFFRFAALKGLK-QG
C.sativus          43  ASISVDDVNHPR-----------Q---IRSSPRFSGIDKTVIESLPFFRFSTLKGTK-EG
Cucumis            64  ASISVDGVNHPR-----------Q---IRSSSRFSGIDKTVIESLPFFRFSTLKGTK-EG
Medicago           69  QLLPISDDPNNQ-----------LATLMRSRSRFSGIDKTAIESLPFFKFSSLKGSK-QG
Glycine            66  ASSPVGDTENQL-----------PF--VRSRSRFSGIDKNVIESLPFFRFSSLKGSK-EG
consensus         181                                  . ..... ............... . ..
consensus          61                                   . .   .                  ..
```

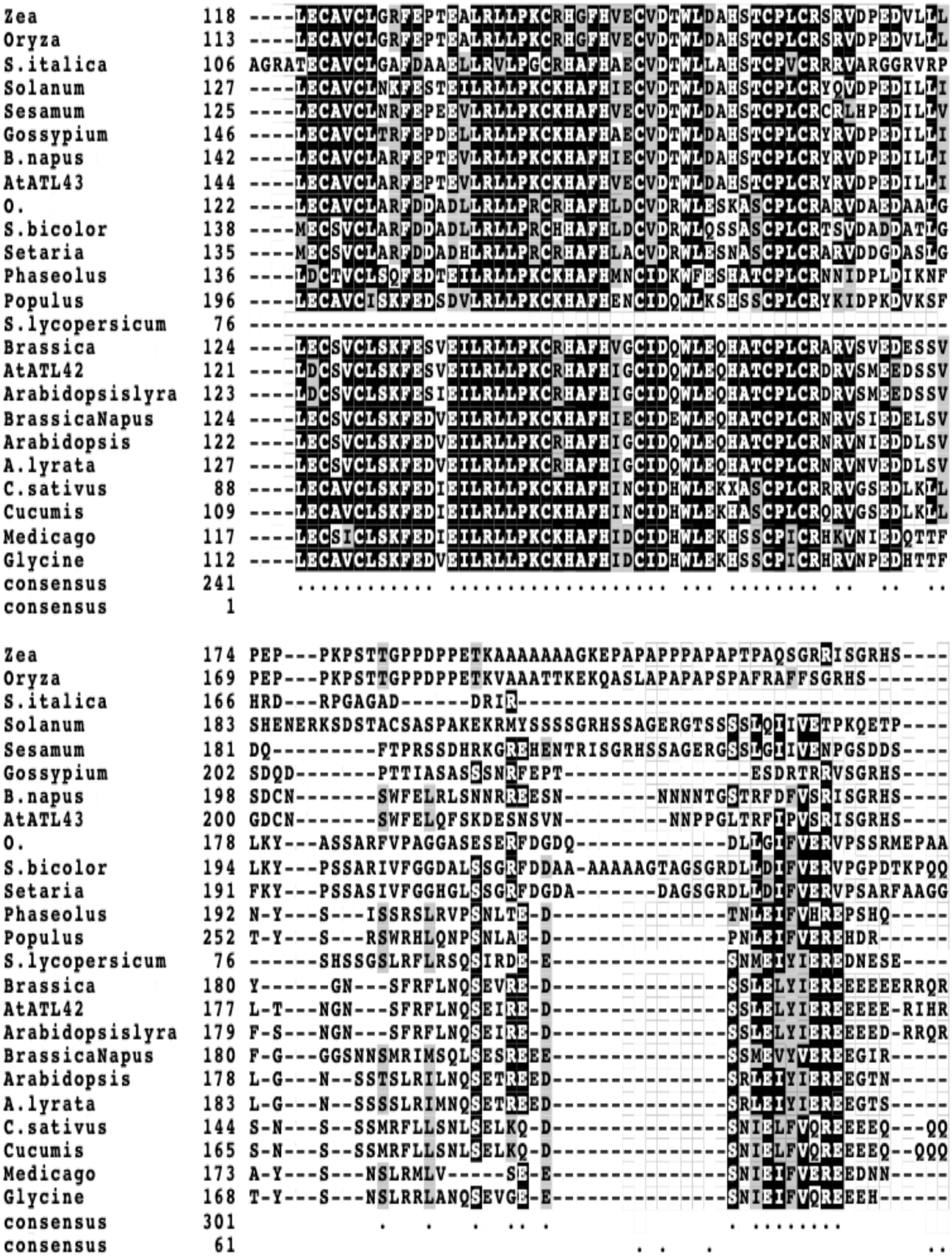
Zea 118 ----LECAVCLGRFEPTEALRLLPKCRHGFHVECVDTWLDAHSTCPLCRSRVDPEDVLLL
Oryza 113 ----LECAVCLGRFEPTEALRLLPKCRHGFHVECVDTWLDAHSTCPLCRSRVDPEDVLLL
S.italica 106 AGRATECAVCLGAFDAAELLRVLPGCRHAFHAECVDTWLLAHSTCPVCRRRVARGGRVRP
Solanum 127 ----LECAVCLNKFESTEILRLLPKCKHAFHIECVDTWLDAHSTCPLCRYQVDPEDILLI
Sesamum 125 ----LECAVCLNRFEPEEVLRLLPKCKHAFHVECVDTWLDAHSTCPLCRCRLHPEDILLV
Gossypium 146 ----LECAVCLTRFEPDELLRLLPKCKHAFHAECVDTWLDAHSTCPLCRYRVDPEDILLI
B.napus 142 ----LECAVCLARFEPTEVLRLLPKCKHAFHIECVDTWLDAHSTCPLCRYRVDPEDILLI
AtATL43 144 ----LECAVCLARFEPTEVLRLLPKCKHAFHVECVDTWLDAHSTCPLCRYRVDPEDILLI
O. 122 ----LECAVCLARFDDADLLRLLPRCRHAFHLDCVDRWLESKASCPLCRARVDAEDAALG
S.bicolor 138 ----MECSVCLARFDDADLLRLLPRCHHAFHLDCVDRWLQSSASCPLCRTSVDADDATLG
Setaria 135 ----MECSVCLARFDDADHLRLLPRCRHAFHLACVDRWLESNASCPLCRARVDDGDASLG
Phaseolus 136 ----LDCTVCLSQFEDTEILRLLPKCKHAFHMNCIDKWFESHATCPLCRNNIDPLDIKNF
Populus 196 ----LECAVCISKFEDSDVLRLLPKCKHAFHENCIDQWLKSHSSCPLCRYKIDPKDVKSF
S.lycopersicum 76 --
Brassica 124 ----LECSVCLSKFESVEILRLLPKCRHAFHVGCIDQWLEQHATCPLCRARVSVEDESSV
AtATL42 121 ----LDCSVCLSKFESVEILRLLPKCRHAFHIGCIDQWLEQHATCPLCRDRVSMEEDSSV
Arabidopsislyra 123 ----LDCSVCLSKFESIEILRLLPKCRHAFHIGCIDQWLEQHATCPLCRDRVSMEEDSSV
BrassicaNapus 124 ----LECSVCLSKFEDVEILRLLPKCKHAFHIECIDEWLEQHATCPLCRNRVSIEDELSV
Arabidopsis 122 ----LECSVCLSKFEDVEILRLLPKCRHAFHIGCIDQWLEQHATCPLCRNRVNIEDDLSV
A.lyrata 127 ----LECSVCLSKFEDVEILRLLPKCRHAFHIGCIDQWLEQHATCPLCRNRVNVEDDLSV
C.sativus 88 ----LECAVCLSKFEDIEILRLLPKCKHAFHINCIDHWLEKXASCPLCRRRVGSEDLKLL
Cucumis 109 ----LECAVCLSKFEDIEILRLLPKCKHAFHINCIDHWLEKHASCPLCRQRVGSEDLKLL
Medicago 117 ----LECSICLSKFEDIEILRLLPKCKHAFHIDCIDHWLEKHSSCPICRHKVNIEDQTTF
Glycine 112 ----LECAVCLSKFEDVEILRLLPKCKHAFHIDCIDHWLEKHSSCPICRHRVNPEDHTTF
consensus 241
consensus 1
Zea 174 PEP---PKPSTTGPPDPPETKAAAAAAAGKEPAPAPPPAPAPTPAQSGRRISGRHS----
Oryza 169 PEP---PKPSTTGPPDPPETKVAAATTKEKQASLAPAPAPSPAFRAFFSGRHS-------
S.italica 166 HRD---RPGAGAD------DRIR-------------------------------------
Solanum 183 SHENERKSDSTACSASPAKEKRMYSSSSGRHSSAGERGTSSSSLQIIVETPKQETP----
Sesamum 181 DQ---------FTPRSSDHRKGREHENTRISGRHSSAGERGSSLGIIVENPGSDDS----
Gossypium 202 SDQD-------PTTIASASSSNRFEPT----------------ESDRTRRVSGRHS----
B.napus 198 SDCN-------SWFELRLSNNRREESN--------NNNNTGSTRFDFVSRISGRHS----
AtATL43 200 GDCN-------SWFELQFSKDESNSVN---------NNPPGLTRFIPVSRISGRHS----
O. 178 LKY---ASSARFVPAGGASESERFDGDQ-------------DLLGIFVERVPSSRMEPAA
S.bicolor 194 LKY---PSSARIVFGGDALSSGRFDDAA-AAAAAGTAGSGRDLLDIFVERVPGPDTKPQQ
Setaria 191 FKY---PSSASIVFGGHGLSSGRFDGDA-------DAGSGRDLLDIFVERVPSARFAAGG
Phaseolus 192 N-Y---S---ISSRSLRVPSNLTE-D---------------TNLEIFVHREPSHQ-----
Populus 252 T-Y---S---RSWRHLQNPSNLAE-D---------------PNLEIFVEREHDR------
S.lycopersicum 76 ------SHSSGSLRFLRSQSIRDE-E---------------SNMEIYIEREDNESE----
Brassica 180 Y------GN---SFRFLNQSEVRE-D---------------SSLELYIEREEEEEERRQR
AtATL42 177 L-T---NGN---SFRFLNQSEIRE-D---------------SSLELYIEREEEE-RIHR
Arabidopsislyra 179 F-S---NGN---SFRFLNQSEIRE-D---------------SSLELYIEREEED-RRQR
BrassicaNapus 180 F-G---GGSNNSMRIMSQLSESREEE---------------SSMEVYVEREEGIR-----
Arabidopsis 178 L-G---N--SSTSLRILNQSETREED---------------SRLEIYIEREEGTN-----
A.lyrata 183 L-G---N--SSSSLRIMNQSETREED---------------SRLEIYIEREEGTS-----
C.sativus 144 S-N---S--SSMRFLLSNLSELKQ-D---------------SNIELFVQREEEEQ---QQ
Cucumis 165 S-N---S--SSMRFLLSNLSELKQ-D---------------SNIELFVQREEEEQ--QQQ
Medicago 173 A-Y---S---NSLRMLV-----SE-E---------------SNIEIFVEREEDNN-----
Glycine 168 T-Y---S---NSLRRLANQSEVGE-E---------------SNIEIFVQREEEH------
consensus 301
consensus 61

```
Zea              227 T-GSVRAPGRVGP----------ASRRSA-----DGGVAVGCFDGAKVRKDRVLLVEPAA
Oryza            219 T-GSVRAPGRVGP----------ASRRSADLVGGDGDAAVGCFEAAKVRKDRVLLMEPAA
S.italica            ------------------------------------------------------------
Solanum          239 SFLNKRMSLDSWN----------FYRKKSKSTNST--------SLSRKD--GMLLSKKKA
Sesamum          228 ---RGRISLDSWR----------SRRKTGNPASENKGRSSGWKSSRKD--GPVSDSSTA
Gossypium        235 YAAGERT------------------------------------------------AAATG
B.napus          239 SAGERASRLNEVRKFS-------SFRRSLDSSLRINDGEEEKTDSVAVAVGCFDRNQQRK
AtATL43          240 SAGERASRLNEIRTSSSYKSNPMSFRRSLDSSLKVNDAGEEKSESVAVNCLDR--L-QRK
O.               222 ------------------------------------------------------------
S.bicolor        250 ------------Q----------QQQQVGDVDD-----------------EEEAISSSVA
Setaria          241 ------------A----------GPKQQAD-----------------------EEAASAR
Phaseolus        224 -GSSSSSRFNIGS----------RFWNLSSSSR------------------KKLLVDQEV
Populus          283 ---QVSSCFNPGS----------SFQISNDNSK-----------------K-EELLVQAG
S.lycopersicum   110 ----------------------------------------------------ELL-----
Brassica         215 EELGGSSRFSIGG----------SFRKILKLGH-----------------KDKPLLDQHG
AtATL42          213 EELSGSSRFSIGE----------SFRKILKLGN-----------------KEKTLLDEHV
Arabidopsislyra  215 DELGGSSRFSIGE----------SFRKILKLGN-----------------KEKTLLDEHG
BrassicaNapus    216 ---DGSSRY---S----------SFRKILNFGK---------------------NDNSLS
Arabidopsis      212 ---DGSSRF---S----------SFRKILKKSL---------------------LLEREG
A.lyrata         217 ---DGSSRF---S----------SFRKILKKSL---------------------LLEREG
C.sativus        179 QILHGSSRFSIGR----------SFRKILKNDK-----------------ENEMLISKAS
Cucumis          201 QILHGSSRFSIRR----------SFRKILKNDK-----------------ENEMLIPQAS
Medicago         200 --QHGSSRFSIGS----------SFRKIGKATTIFKEEEKGIISK-----EEELLIEK--
Glycine          199 ---HGSSRFSFGS----------SFRKTGKYVK-----------------EEEFLIEKGA
consensus        361      ....              ....                          ..    .
consensus          1

Zea              271 VV--AEPDPVAFDRRFGHRILVSTAGGCKGETAPAAQQRWSDLRPSDLMFVRAEFLVTDA
Oryza            268 AV--AEPDPEAFDRRFGHRILVSTAGGCEDETAPAAKQRWSEVRPSDLMFVRSEMLVTEA
S.italica            ------------------------------------------------------------
Solanum          279 PAPATEP--EVEDRRLEHRIIISGDEPELDTA-SGSRQRWSDVEACDRLYLRSEMLLSES
Sesamum          273 SRKDGQLPVQGDGHRLEHRIIISGAGEAEEEE-G---RRWSDVQASEILYLRSEMILGGE
Gossypium        247 GSY---RKDGMLLKRMEHRIIVSGTGT----E-SGYQQRWSDVQPSDLLYLRSEMIISQS
B.napus          292 DGLLLNSNRESFEARFEHRIIISGGN---------RDQRWSEVRPADLLYLRSEMILSDC
AtATL43          297 DGLLLIPNRESFEGRFEHRIIISGGN--------RDDQRWSEVRPSDLLYLRSEMILSDC
O.               222 A------AHCPDLDRYKHRIVVSDAV---------FKSRWSEINSSDLIALDTELLRSME
S.bicolor        271 L------PPGQELDRHKHRIIVSDVV---------FKSRWSELNSADLIALDMEMLRSMS
Setaria          256 A------PPSPELDRHKHRIIVSDVV---------FKSRWSELNSADLIALDTEMLRSMS
Phaseolus        255 SG-----TNGTHVHKFYHKIVVSDVV---------RRSRWSDLNSSDMLSLKSEMIHHVS
Populus          312 GN---ADDNRKLFHKFMHKIIISDVL---------IKSRWSDANSSDFLSLNTEMLGVMS
S.lycopersicum   113 -------IQGKALDRFNHRIIVSDVV---------FKNRWSNVTSSDLIFLNSEMLNGIT
Brassica         248 E--------DKAMHKFNHRILVSDVV---------FKNRWSNVSSSDLMFLNSEMVSSIS
AtATL42          246 N----DKDEKKLMHKFNHRIVVSDVV---------FKNRWSNVSSSDLMFLNSEMVNSIS
Arabidopsislyra  248 N---SDKDEKKLMHKFNHRIVVSDVV---------FKNRWSNVSSSDLMFLNSEMVNSIS
BrassicaNapus    239 L---VEQGNEKCLHKFNHRIVVSDAV---------FKNRWSNVTPADLTFLTSNMLSSTS
Arabidopsis      235 N---ENIDEKKLMHKFNHRIVVSDAV---------FKNRWSNITSSDLTFLTSEMLNSVS
A.lyrata         240 N---ENIDEKKLMHKFNHRIVVSDVV---------FKNRWSNVTSSDLTFLTSEMLNSLS
C.sativus        212 GD--YEDEKMKNLHRHNHKIIVSDFV---------FMNRWSNVSSSDLMFLNKEMIDAIS
Cucumis          234 CD--YEDEKMKNLHRHNHKIIVSDFV---------FMNRWSNISSSDLMFLNKEMIDAIS
Medicago         241 ----SD-SNNKAYHKHNHKITISDVV---------FKHRWSNVSSSDLMFLNSEMINASS
Glycine          229 ED--SD-GNQKGYHKHNHRITISDVV---------FKHRWSNVSSSDLMFLNSEMLNDAS
consensus        421           . ....  .......  .         ........ ......  ....  ...
consensus         61                                 . .   .                      ..
```

```
Zea              329 ---GRHSCSAA-------VNS---------------------------------------
Oryza            326 ---GRYSCSAA-------VHS---------------------------------------
S.italica            ------------------------------------------------------------
Solanum          336 ---RRYSGSRKR------TAE---------------------------------------
Sesamum          329 ------------------------------------------------------------
Gossypium        299 ---RRLRI----------------------------------------------------
B.napus          343 ---RKLAA----------------------------------------------------
AtATL43          349 ---RKLAA----------------------------------------------------
O.               267 TMEVEMEMELYKETEEEETDHDHQRKTLLAASGGRSSVDAAA-------RARMISS----
S.bicolor        316 SSSGRFVFPPDSPYCPEYSNEAQQAPNNKEIAAEEEEEEDEDGVVPTTSRKRLLKPVVVV
Setaria          301 --SGRFSFPDYSPEYNEAKLSLSAAGEEEAYGAVPTTTGETE-------RKRLLVDGRSG
Phaseolus        301 --TGRFSPSNEF------F-----CGNSSLSSIFNADES---------------------
Populus          360 --SNRFTPLKSS------S--ARFYNGLSRVENLEKVKDDTERKRLSKPQFPTVDRSNSV
S.lycopersicum   157 --SNRFSSMDTT------CEQSRVIE----------------------------------
Brassica         291 --SERFSSMDRV------K-----RGDEEDQRGNVGIKEEKGMLEN--------------
AtATL42          293 --SERFSSLDHV------K-----RGDEEDQIGILRIKEEMEAKRMLENKLTSMTTM---
Arabidopsislyra  296 --SERFLSIDRA------K-----RGDEEDQIGILRIKEEMEAKRMLENKLTSMKTM---
BrassicaNapus    287 --SDRFSSIDRV------Q-----RGDL-------RDKEEIDMKKMVDN-----------
Arabidopsis      283 --SDRFSSVDRV------H-----RGNL-------RDKEDMEMKRMLIKH----------
A.lyrata         288 --SDRFSSMDRV------H-----RGNL-------RDKEDMEMKRMLKKN----------
C.sativus        261 --SRRFTSLETD------IEQ-STLPRSRQIEEILKIKEEMEIKRSFESKLNKKPKA---
Cucumis          283 --SCRFTSLDTD------IEQ-STLPRSMQIDEILKIKEEMEIKRSFESKLNTKIQSNSI
Medicago         287 --SNRFNNMESK------F-----------------------------------------
Glycine          277 --SNRFSNLESN------ADMMSSTRGVVENEQIMNIKEEMERKISFESKVGVLNNIKSV
consensus        481   . ... ..                            ..
consensus          1

Zea              340 ------------------GNARSAIGVRSVSELAGVRRLPPI------------------
Oryza            337 -----------------SSNGRSVISARSLSELAGVSRLPPI------------------
S.italica            ------------------------------------------------------------
Solanum          348 -----------------MESGRRVINERSVSELTGMSRFKSN------------------
Sesamum          329 -----------------EGSGRGEINERSVSEITGVSRYRNN------------------
Gossypium        304 -----------------GGALVAETGNDDNN-----KNGNG-------------------
B.napus          348 -----------------AEDGREVIGGRSVSELTSIDRRRR-------------------
AtATL43          354 -----------------AEGGRDVINGRSVSELTGIERRRR-------------------
O.               316 --------------------SSSSGSVRSMSEMVSLPRLRAA------------------
S.bicolor        376 DARLGGGCSSGSAVSASA-ARMISSGVRSMSEIVSLPRLRAA------------------
Setaria          352 GGRCSSSAAVDAAVPAA--ARMISSGVRSMSEIVSLPRLRGA------------------
Phaseolus        327 -----------------SLTLLNTAEKRSMSDIAHVPRFIERCEQN--------------
Populus          410 PSSSLNSS-----------KMLNPVGKRSTSEITIFSRFRQL---------------SAK
S.lycopersicum   175 ----------------EDQSINIKDEKRSMSEIIVYSRFNNR------------------
Brassica         324 ----KLSENRDFGSKSR--NVMIEPGRRSISDITAVPRLSVV-------VHGDCSGSNAA
AtATL42          337 ----FSSENGDSGSKSR--SVMIEPGRRSVSDITAVPRLSIS-------IHGDCSGSAAE
Arabidopsislyra  340 ----FSPENGDSGSKSR--SVMIEPGRRSVSDITAVPRLSIS-------VHRDCSGSAAG
BrassicaNapus    316 ----------------------KDSTRRSVSEITAVPRLSVVTTAARENAVGGSNSGLDA
Arabidopsis      313 ----------------------KDSSRRTVSEITTVSREKA--------VGGSYRGST--
A.lyrata         318 ----------------------KDTSRRTVSEIMTVSRENA--------VGGSYSGSTAA
C.sativus        309 ------------------------------------------------------------
Cucumis          334 LGYPSTSQSSTNPSQTTR--ITSPDARRSVSEITGISRFGHD--------DDLYMNFNRK
Medicago         298 --------------------QDYDGEKRSVSEITGVSRFKDF--------D------MKK
Glycine          329 SDKHLLFTSDSAGKSTHAPKYANPGEKRSMSEITAVSRFGDL--------G------MKM
consensus        541                           .......... .
consensus         61                                  . .   .                  ..
```

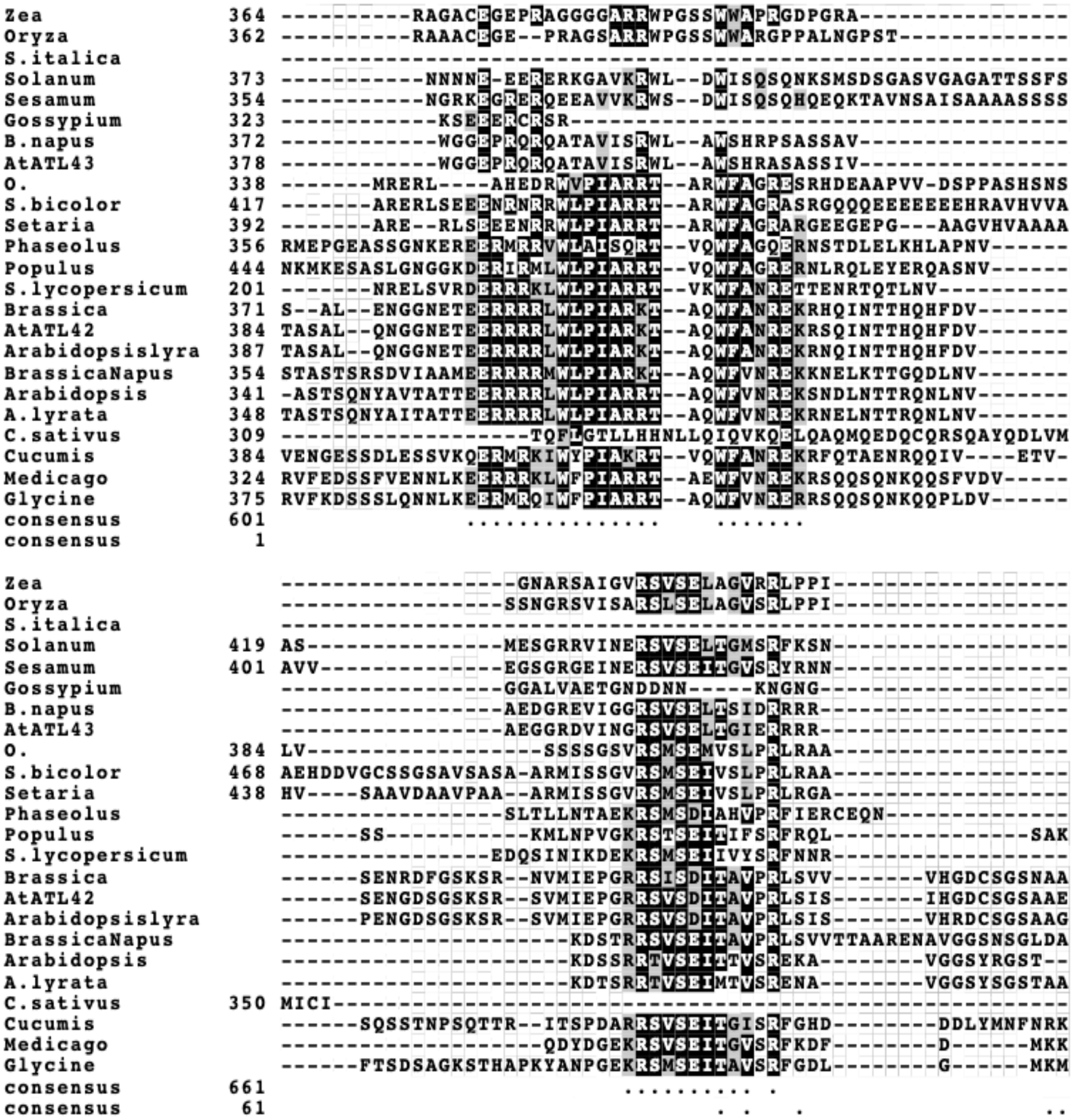

Supplemental Figure S1. ATL12 homologs protein alignment.
