# Supplementary material for: Arabidopsis Toxicos en Levadura 12 Modulates Salt Stress and ABA Responses in Arabidopsis thaliana
Source: Int J Mol Sci. 2022 Jun 30;23(13):7290. doi: 10.3390/ijms23137290 (PMC9266925; doi:10.3390/ijms23137290)
Supplement: Supplementary file 1 [file ijms-23-07290-s001.zip › Supplemental Figure S2.pdf]

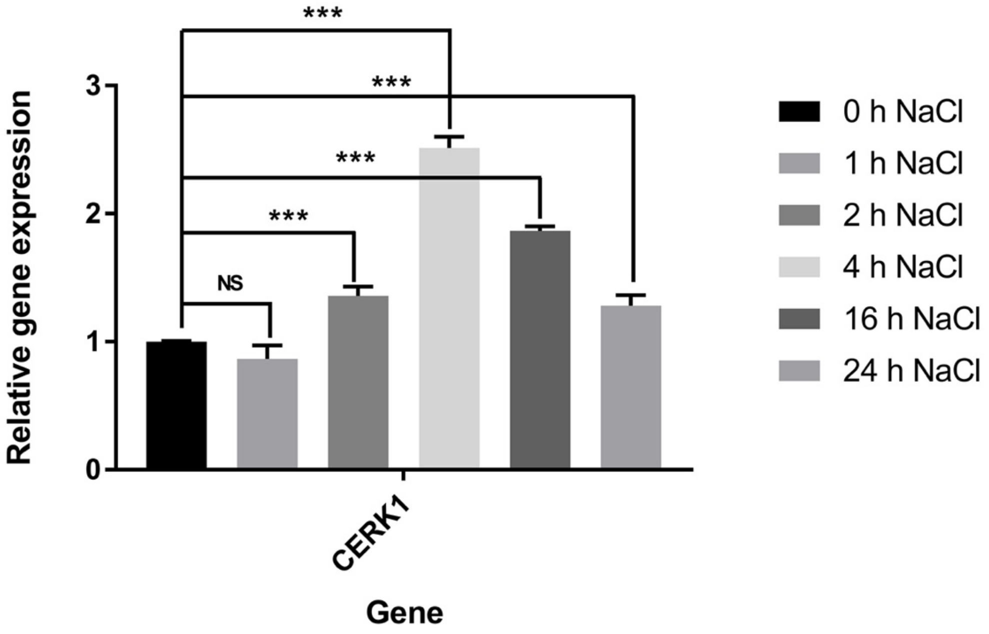

Supplemental Figure S2. qRT-PCR analysis of *CERK1* expression in *Col-0* in response to salt stress. Asterisks indicate statistically significant differences between the samples, according to the One-way ANOVA analysis and multiple comparison post Tukey's test. **** indicates $p < 0.0001$, *** indicates $p < 0.001$, and NS indicates not significant. The black line indicates that the significant differences are present between datasets. The error bar indicates means ± the SD of three independent biological replicates.
